# A species assemblage approach to comparative phylogeography of birds in southern Australia

**DOI:** 10.1002/ece3.87

**Published:** 2012-02

**Authors:** Gaynor Dolman, Leo Joseph

**Affiliations:** 1 Australian National Wildlife Collection, CSIRO Ecosystem SciencesGPO Box 1700, Canberra, ACT 2601, Australia; 2 Australian Centre for Evolutionary Biology and Biodiversity, School of Earth and Environmental Sciences, The University of AdelaideSouth Australia 5005, Australia

**Keywords:** Comparative phylogeography, IMa, MsBayes, Nullarbor, statistical phylogeography

## Abstract

We present a novel approach to investigating the divergence history of biomes and their component species using single-locus data prior to investing in multilocus data. We use coalescent-based hierarchical approximate Bayesian computation (HABC) methods (MsBayes) to estimate the number and timing of discrete divergences across a putative barrier and to assign species to their appropriate period of co-divergence. We then apply a coalescent-based full Bayesian model of divergence (IMa) to suites of species shown to have simultaneously diverged. The full Bayesian model results in reduced credibility intervals around divergence times and allows other parameters associated with divergence to be summarized across species assemblages. We apply this approach to 10 bird species that are wholly or patchily discontinuous in semi-arid habitats between Australia's southwest (SW) and southeast (SE) mesic zones. There was substantial support for up to three discrete periods of divergence. HABC indicates that two species wholly restricted to more mesic habitats diverged earliest, between 594,382 and 3,417,699 years ago, three species from semi-arid habitats diverged between 0 and 1,508,049 years ago, and four diverged more recently, between 0 and 396,843 years ago. Eight species were assigned to three periods of co-divergence with confidence. For full Bayesian analyses, we accounted for uncertainty in the two remaining species by analyzing all possible suites of species. Estimates of divergence times from full Bayesian divergence models ranged between 429,105 and 2,006,355; 67,172 and 663,837; and 24,607 and 171,085 for the earliest, middle, and most recent periods of co-divergence, respectively. This single-locus approach uses the power of multitaxa coalescent analyses as an efficient means of generating a foundation for further, targeted research using multilocus and genomic tools applied to an understudied biome.

## Introduction

Environmental change during the Plio-Pleistocene had a major influence on the composition and distribution of species globally (e.g., Hewitt 1996, 2004; Avise and Walker 1998; Provan and Bennett 2008). Northern hemisphere ice sheets made vast regions uninhabitable and caused the majority of species there to persist only in southern refugia (Provan and Bennett 2008). In contrast, Australia was not glaciated during this period, but its biota still faced environmental transformations associated with glacial cycles. The continent's Miocene mesic climates and environments (e.g., Kershaw et al. 1994; Martin 2006) became the more xeric ones that dominate the continent today (McLaren and Wallace 2010). Pleistocene glacial cycles caused repeated expansion and contraction of the Australian arid zone culminating in the most severe arid phase at the Last Glacial Maximum (20–18 kA) (Williams et al. 2009). Consequently, some species and lineages once presumably more widespread across southern Australia are now restricted to the southwest (SW) and southeast (SW) or east of the continent. A sample of such histories is our focus in this paper.

Relatively few Australian biogeographic regions and barriers have been studied using comparative phylogeography (Avise 2000). Key examples, however, mostly apply to eastern Australian tropical and sclerophyllous forest environments (e.g., Joseph et al. 1995; Hugall et al. 2002; Schauble and Moritz 2001; Stuart–Fox et al. 2001; Garrick et al. 2008). Comparative phylogeographies of arid and semi-arid zone bird species have revealed variable levels of phylogeographic structure, some species lacking structure (Joseph and Wilke 2007) and others showing clear signals of historical isolation (Joseph and Wilke 2006; Joseph and Omland 2009; Kearns et al. 2009). Concerning studies of major phylogeographic barriers to gene flow in particular, the Carpentarian Barrier in northern Australia (Jennings and Edwards 2005; Lee and Edwards 2008) and the Black Mountain Corridor in the Wet Tropics rainforest (Dolman and Moritz 2006) have additionally been studied with multilocus approaches. Together, phylogeographic studies of single species, barriers, and regions, whether from single locus or multilocus data, are bringing new dimensions to understanding Australia's Plio-Pleistocene biogeography (Byrne 2008; Byrne et al. 2008, 2011). Nonetheless, some biogeographic regions have been under-represented. In these regions, a single-locus comparative phylogeographic approach using the power of multitaxa coalescent analyses is an efficient way of providing sound foundational knowledge for future multilocus studies.

Limitations of mitochondrial DNA (mtDNA) as an inferential tool in phylogeography are well known. They arise from stochastic variation in the coalescent process (Kuo and Avise 2005), selection (Ballard and Whitlock 2004), and sex bias in fitness or dispersal behavior (Hare 2001). These limitations can be overcome by surveying more loci (Edwards and Beerli 2000) or more species using comparative phylogeography (Avise 2000). A remaining problem with comparative phylogeography is that signatures of disparate timing of divergence may reflect either continuous divergence, multiple discrete periods of divergence, or stochastic variation in the coalescent or differences in population demography around a simultaneous divergence (e.g., Dolman and Moritz 2006; Hickerson et al. 2006). Further, a single locus offers low precision for estimating parameters such as population divergence time (Edwards and Beerli 2000; Hudson and Turelli 2003). A solution to this conundrum comes from recently developed coalescent-based methods (Hickerson et al. 2006) that make it possible to re-examine the utility of mtDNA alone in testing for temporal congruence among species across a putatively common barrier. These methods can account for demographic variation and coalescent stochasticity and estimate the number and timing of periods of co-divergence among a suite of species affected by the same barrier. Further, they can exploit variation between species with identical histories of divergence to estimate divergence time (akin to variation between loci within a single species divergence).

Coalescent-based hierarchical approximate Bayesian computation (HABC) methods for estimating the number and timing of periods of co-divergence have shed light on a number of well-recognized barriers to gene flow (Beaumont 2010). For simplicity, hereafter, these discrete episodes when multiple species co-diverged will be referred to as “co-divergence events.” Two historical co-divergence events were inferred across both the Isthmus of Panama (Hickerson et al. 2006) and Baja California's mid-peninsula (Leaché et al. 2007). Likewise, comparative mtDNA phylogeographies of hydrothermal species in the East Pacific Rise (Plouviez et al. 2009), birds of the Queen Charlotte Islands (Topp and Winker 2008), and across the Isthmus of Tehuantepec (Barber and Klicka 2010) have also benefited from the statistical rigor of coalescent-based HABC using MsBayes (Hickerson et al. 2006, 2007). Most recently, Ilves et al. (2010) demonstrated the advantages of HABC analyses in testing long-standing hypotheses of colonization or persistence of intertidal and subtidal invertebrate communities of the west and east coasts of the Atlantic Ocean.

**Figure 1:**
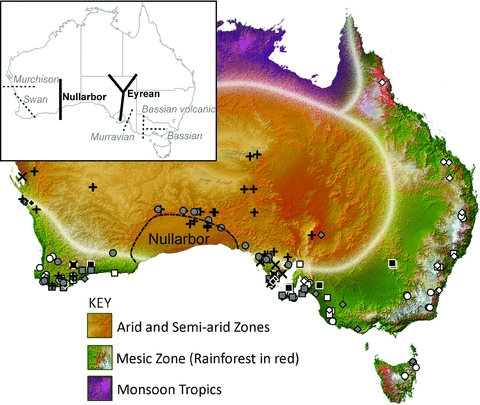
Map of Australia showing locations of Eyrean and Nullarbor Barriers in relation to other putative, minor barriers in southern Australia (after Schodde and Mason 1999), and sampling localities for 10 bird species. Mesic species: white circles = *Petroica boodang*, white squares = *Phylidonyris novaehollandiae*, white diamonds = *Melithreptus lunatus*; semi-arid zone species: gray diamond = *Gliciphila melanops*, black square (white outline) = *Drymodes brunneopygia,* gray square = *Glossopsitta porphyrocephala*, black x = *Malurus pulcherrimus,* black diamond (white outline) = *Eopsaltria griseogularis*, black cross = *Malurus splendens*, gray circle = *Climacteris rufus*.

Here, we apply these methods to study the Plio-Pleistocene histories of 10 bird species that are either isolated in mesic zones of Australia's SE and SW or which are patchily discontinuous in semi-arid habitats between those regions. Several putative barriers to gene flow have been recognized in this broad biogeographic region (see Fig. 1, inset) (Schodde and Mason 1999). The Lake Eyre Basin/Eyrean Barrier (Keast 1961; Ford 1974; Schodde 1982) has been affirmed by molecular data as a major phylogeographic break in Australian ringneck parrots (*Barnardius zonarius*) (Joseph and Wilke 2006) and the splendid fairy-wren (*Malurus splendens*) (Kearns et al. 2009). Here, we focus attention on the Nullarbor Plain, which is another barrier of likely pivotal biogeographic importance because of its geographically central location in southern Australia (Fig. 1). The Nullarbor Plain is an extensive region of some 200,000 square kilometers (Webb and James 2006) of limestone karst, formed through elevation of limestone sediments about 14 million years ago (Mya) (Li et al. 2004) and continuously exposed since then (Sheard and Smith 1995). It is dominated by chenopod shrubs. Currently, it extends from the continent's south coast northwards to the southern fringes of the more heavily vegetated central Australian arid zone (see review in McKenzie and Robinson 1987). Coupled with its fringing xeric woodlands and shrublands, it forms the core of a strong biogeographic barrier of approximately 700 km between Australia's temperate mesic zones in the SW and SE (Fig. 1). At glacial maxima, lower sea levels allowed rivers to drain more effectively, resulting in fewer swamps and floodplains (Haq et al. 1987; Macphail et al. 1994). This, together with regression of the shoreline causing subaerial exposure of the Great Australian Bight (Li et al. 2004), offered opportunity for some species to disperse and recolonize, potentially allowing gene flow between the SW and SE.

Crisp and Cook (2007) used relaxed molecular clock dating to estimate the time of interspecific divergences in sister species pairs of plants on either side of the Nullarbor Plain. Divergence times between 23 endemic SW plant lineages and sister lineages endemic to SE were mostly relatively ancient (clustering around 13–14 Mya). Mid-Miocene aridification and/or elevation of the Nullarbor Plain as a vicariant barrier were inferred as the driving agents of these divergences. Three younger divergences of 2–4 Mya were thought to be due to severe drying around this time (Fujioka et al. 2005). Among birds, phylogeographic studies of Australian magpies (*Gymnorhina tibicen*) (Toon et al. 2007), southern emu-wrens (*Stipiturus malachurus*) (Donnellan et al. 2009), and musk ducks (*Biziura lobata*) (Guay et al. 2010) have also revealed divergent monophyletic intraspecific lineages on either sides of the Nullarbor Plain and its fringing semi-arid habitats.

In this study, we first test for temporal congruence of divergence across the Nullarbor Plain using HABC. For this, we use the MsBayes software pipeline that allows for variation in stochasticity of the coalescent process and variation in demographic histories among taxa (Hickerson et al. 2006, 2007). We estimate the number of co-divergence events that generated the current distributions of the 10 bird species, assign suites of species to the appropriate co-divergence event and estimate their timing (Hickerson et al. 2006, 2007).

**Figure 2:**
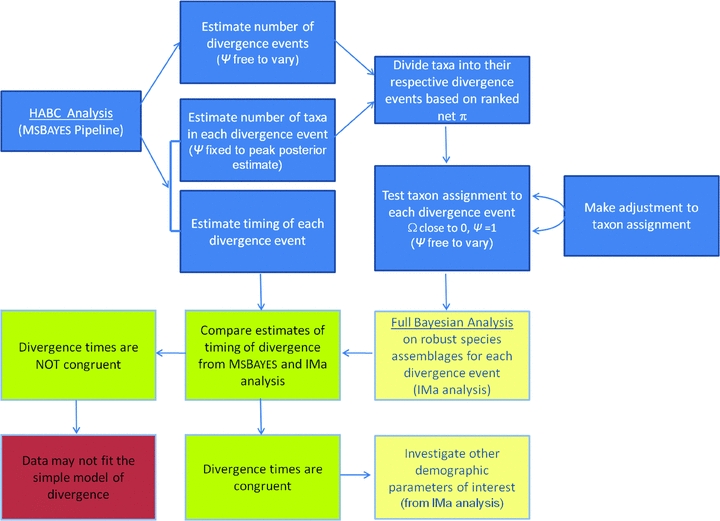
A flow chart outlining the sequence of HABC and Full-Bayesian analyses used in estimating parameters associated with the divergence of species assemblages. Box colors are indicative of the type of analysis: HABC (MsBayes) = blue; full Bayesian (IMa) = yellow; comparison and inference = green or red).

Our approach is summarized in Figure 2 and as follows. For the species assemblage divergence(s) uncovered using the HABC approach, we apply a full Bayesian approach (IMa) (Hey and Nielsen 2007) to estimate population demographic parameters associated with the divergence of suites of species (hereafter termed species assemblage divergence). To our knowledge, this is the first time IMa has been used in a species assemblage context. IMa (Hey and Nielsen 2004, 2007) is intended to fit an Isolation-with-Migration (IM) model to genetic data from multiple loci sampled from a single pair of divergent populations. IMa's goal is to estimate demographic parameters associated with the two populations’ divergence from their ancestral population (Nielsen and Wakeley 2001; Hey and Nielsen 2004). Rather than estimating population-splitting parameters for the SW and SE population pair of each species using multiple loci, we use IMa for a novel purpose. Having first used MsBayes to identify discrete co-divergence events relevant to 10 southern Australian bird species, we then used IMa to estimate demographic parameters associated with each co-diverging species assemblage. Thus, for a given co-divergence event, rather than our approach and data exhibiting stochasticity of the coalescent process among loci, they represent stochasticity across multiple species affected by the same co-divergence event. IMa allows inheritance scalars (*h*) to be estimated as parameters in the model, such that *h* for each locus is free to vary during the course of the Markov chain as a function of the data and the model. There are two biological reasons for allowing *h* to vary among loci in IM (Hey and Nielsen 2004): first to account for biases in the effective sex ratio of males and females, and second the effective population size of particular loci could be reduced relative to other loci in the genome through recurrent selective sweeps (Maynard Smith and Haigh 1974; Gillespie 2000) or background selection (Charlesworth et al. 1993). In the context of running IMa as a taxon assemblage divergence model, invoking the option to estimate *h* allows the model to account for differences (within reason) in effective population sizes among 10 SW and SE population pairs of southern Australian birds. In this context, estimating *h* allows effective population sizes to differ between species rather than between separate loci within a species. A limitation, however, is that effective population sizes of ancestral and the two divergent populations are not able to vary idiosyncratically with respect to each other across all species. Effective population sizes of each of SW and SE population pair and their ancestral population are thus assumed to vary consistently across all taxa. This may be a considerable assumption if species have responded to habitat fluctuations differently.

## Materials and Methods

### Sampling

We sampled four southern Australian mesic zone bird species that have populations isolated in SW and SE (taxonomy and nomenclature follows Christidis and Boles 2008): scarlet robin (*Petroica boodang*), new holland honeyeater (*Phylidonyris novaehollandiae*), white-naped honeyeater (*Melithreptus lunatus*), and tawny-crowned honeyeater (*Gliciphila melanops*). We sampled six species of semi-arid southern Australia, each having populations isolated wholly or partially in SW and SE: purple-crowned lorikeet (*Glossopsitta porphyrocephala*), southern scrub-robin (*Drymodes brunneopygia*), blue-breasted fairy-wren (*M. pulcherrimus*), western yellow robin (*Eopsaltria griseogularis*), splendid fairy-wren (*M. splendens*), and rufous treecreeper (*Climacteris rufus*), the last two being patchily continuous in semi-arid and arid habitat to the north of the Nullarbor Plain. Two hundred seventy-eight individuals (*n* = 10–48 per species) were sampled from these 10 species. This included 15 newly acquired specimens of *M. splendens* from the Great Victoria Desert north of the Nullarbor Plain, which had been identified in that species as a major sampling gap, and 32 *ND2* sequences from *M. splendens* (Kearns et al. 2009) downloaded from GenBank (EU144272-EU144303). To focus attention on the Nullarbor Plain and its fringing semi-arid habitats, we excluded samples east of the Eyrean Barrier (Fig. 1) if Fst for a given species was significant across that barrier. Museum specimen and locality details of all individuals studied, and mtDNA genealogies are provided in supplementary material.

### DNA extraction and sequencing

Total cellular DNA was extracted according to the salting-out method of Miller et al. (1988). We amplified the *ND2* gene region of the mtDNA genome using primers L5216 (5′-GGCCCATACCCCGRAAATG-3′) and H6313 (5′-ACTCTTRTTTAAGGCTTTGAAGGC-3′) (http://people.bu.edu/msoren/primers.html). Polymerase chain reactions (PCR) were performed in 25 μl reactions containing 1× reaction buffer (Eppendorf), 0.2 mM dNTP, 0.2 μM each primer, 1 Unit HotMaster™ Taq DNA Polymerase (Eppendorf), which specifically has no requirement for additional MgCl_2_. Cycling conditions included an initial denaturation of 94°C for 2 min followed by a touch-down protocol of 34 total cycles of 94°C denaturation for 30 sec, 62°C–58 °C annealing for 30 sec, 65°C for 45 sec, and a final extension of 65°C for 5 min. PCR products were purified with Ampure magnetic beads (Agencourt), and sequenced directly in both directions on an ABI 3700 PRISM DNA analyzer.

### Summary statistics and tests of assumptions

DNA divergence indices (nucleotide diversity, π; nucleotide divergence, Da) were calculated using DnaSP v5.0 (Librado and Rozas 2009). Addressing concerns about selective neutrality of avian mtDNA diversity (Zink 2005), we tested for neutrality using MK tests (McDonald and Kreitman 1991) in DnaSP v5.0 (Librado and Rozas 2009).

### Analyses of divergence of species assemblages

A flow chart (Fig. 2) outlines the sequence of HABC and Full-Bayesian analyses used in estimating parameters associated with the divergence of species assemblages. Individual analyses are described in more detail below.

### Tests for temporal congruence of divergence

Hierarchical Bayesian model tests under an ABC framework implemented in the MsBayes software pipeline (Hickerson et al. 2007) tested for temporal congruence of divergence times among populations in SE and SW. The MsBayes software pipeline simultaneously estimates three hyper parameters that quantify the variability in divergence times of the 10 SW and SE population pairs: (1) mean population divergence time (*E[τ]*) in units of μ per generation, (2) degree of discordance (Ω) as the variance of population divergence time *τ*, divided by the mean of *τ* across all population pairs, such that the greater the variance of population divergence time relative to the mean divergence time across all population pairs, the less support there is for simultaneous divergence, and (3) number of discrete co-divergence events (Ψ) (Hickerson et al. 2006). A vector of observed summary statistics is calculated from DNA sequence data of each SW and SE population pair. Random draws of the hyper-parameters and sub-parameters from a coalescent model of population divergence with or without postdivergence gene flow (see Hickerson et al. 2007 for details) were used to simulate finite sites DNA sequence data from 10 population pairs. In the model, ancestral and descendent population sizes are free to vary, thus allowing for the general model of isolation by colonization. From these simulations a vector of summary statistics was produced. From 5,000,000 iterations, acceptance/rejection with regression algorithms were used to compare the observed summary statistics vector with the summary statistics vector from the simulated data. Polychotomous regression was used to estimate Ψ, and local linear regression was used to estimate Ω and *E*(*τ*). Hyper-posteriors were constructed using 1800 accepted draws.

We used the default summary statistics (π, net π, Watterson's θ, and 1/Tajima's D) for acceptance/rejection. We tried a range of upper limits for sub-parameters θ (current population diversity per site per generation) and ancestral population size (relative to current population size) and settled on those that best fit the data (θ upper = 0.025, ancestral population size upper limit = 0.25). We initially tried high upper limit of *τ* = 10. Given the low *τ* estimates from these trials, we reduced the upper bound of *τ* upper to 3.0. Analyses were run with migration rate upper limit set to 0.0, but allowing some migration at migration rate upper limit = 0.5 did not affect results. Recombination rate upper limit was set to 0.0 (assuming no recombination in mtDNA).

HABC analyses were initially performed on the 10 SW and SE population pairs allowing Ψ to vary. For the full Bayesian analysis that follows (see Flowchart; Fig. 2), it is important that the number of discrete co-divergence events is not underestimated, or else multiple divergences will be analyzed as a single event. Overestimation of the number of discrete co-divergence events is less important, except in that it may reduce the power to estimate divergence times. For this reason, the Bayes Factor (BF) was calculated to test the level of support for Ψ≤“highest posterior estimate” versus Ψ > “highest posterior estimate.” BFs were also calculated for the model of simultaneous divergence (Ψ = 1 vs. Ψ > 1) and a model in which diversification is continuous rather than a discrete number of divergence events (i.e., via rare dispersal events across pre-existing geographic or climatic barriers; Ψ <10 vs. Ψ = 10). For tests of simultaneous divergence Ω with local linear regression has been shown to be a reliable indicator (Hickerson et al. 2006). Therefore, BFs were also calculated for Ω = 0 versus Ω > 0.0. The level of support was determined using the scale suggested by Jeffreys (1961). HABC analyses were then performed with Ψ fixed to the highest posterior estimate of Ψ from the initial analysis. To test the confidence in our assignment of species to each co-divergence event, we ran subsets of taxa allowing Ψ to vary. If the hyper-parameter estimate of Ω is very close to zero and Ψ = 1, there is support for a single co-divergence event for that subset of taxa. BFs were calculated for Ψ = 1 versus Ψ >1 to give an indication of the level of support (Jeffreys 1961).

### Estimating demographic parameters of community divergence

IMa (Hey and Nielsen 2004, 2007) and our approach to its use for divergence of species assemblages are described above (see Introduction). To reiterate briefly, the goal is to estimate demographic parameters associated with the divergence of two daughter populations from an ancestral population (Hey and Nielsen 2004; Nielsen and Wakeley 2001). The parameters are scaled by μ, the geometric mean across loci of mutation rate/year/locus and are effective population diversity for ancestral population, θA, and pairs of daughter populations since divergence θ1 and θ2 (θ = 4 Nμ), directional migration rates, *m*1 and *m*2, and population divergence time, *t*.

The data were divided into datasets representing discrete species assemblage divergences as uncovered with MsBayes, and each species assemblage divergence was analyzed separately. Infinite sites and HKY are the only models of nucleotide evolution available for DNA sequence data in the IMa program. We used the HKY model because mtDNA loci are assumed to be nonrecombining and the data do not fit the infinite sites model. Preliminary metropolis Monte Carlo Markov chain simulations (20 million steps) were required to optimize the number of chains, heating model, heating parameters, and upper bounds for the parameter priors. Broad parameter limits were subsequently refined. Six chains with geometric heating (g1 = 0.8, g2 = 0.92) were found to maintain a sufficient level of swapping between chains (between chains 4 and 5 was 5–79% and between chains 0 and 1 was 81–92%). Two final simulations for each divergence were carried out with different random seeds, 50 million steps, and burn-in of five million. Chain swapping and genealogy, branching, and mutation rate mixing were sufficient, there were no trends present in parameter trend plots and effective sample sizes were ample (minimum ESS per run were between 72 and 523). Two final runs per analysis confirmed convergence upon comparable parameter distributions.

### Converting divergence time estimates to a demographic scale

To convert divergence time estimates to a demographic scale, we applied an imported mutation rate based on a general avian divergence rate of 2.1% (Weir and Schluter 2008). The general avian mutation rate was generated with a time reversible (GTR+Γ) model and was cross-validated with 74 avian molecular clock calibrations generated from fossil and biogeographic events across 12 taxonomic avian orders and over the last 12 Ma (Weir and Schluter 2008). Divergence time estimates from MsBayes were calculated using population divergence time in years, t = (*E[τ]*) × (θAve/μ), where (*E[τ]*) is the mean population divergence time in units of μ per generation, θAve is half of the upper bound of the θ prior used in the MsBayes analysis, and μ is the per site per generation mutation rate. We assumed an average generation time of two years based on published data (Higgins 1999; Higgins et al. 2001; Higgins and Peter 2002) and acknowledge that divergence time estimates will be sensitive to assumptions of generation time. Divergence time estimates from IMa were calculated using population divergence time t = *t*/μ, where *t* is the divergence time parameter estimate from IMa, and μ is per locus per year mutation rate. These divergence times were compared to those calculated using the imported mutation rate applied directly to average net divergence (Da) estimates. General patterns of effective population sizes for SW, SE, and ancestral populations are described for each co-divergence event.

## Results

Two hundred forty-six *ND2* sequences were collected from the 10 species. *ND2* sequences did not contain stop codons or indels, consistent with not having amplified pseudogenes. All sequences were submitted to GenBank (JQ27369-JQ27614; see Table S1 for individual level identification).

### Summary statistics and tests of assumptions

Haplotype diversity ranged from 0.739 to 0.963 (Table 1). Nucleotide diversity (π) within SW or SE populations varied by an order of magnitude, from 0.049% in *E. griseogularis* (SW) to 0.54% in *G. melanops* (SW) (Table 2). Five of the 10 population pairs are reciprocally monophyletic, having Da between 0.19% and 4.35% (Table 2). The other five have net divergences up to three orders of magnitude less, between 0.001% and 0.08%. Three of these show no structure between SW and SE populations and individuals from the SW and SE share haplotypes (*D. brunneopygia*, *G. porphyrocephala*, *M. pulcherrimus*) (Table 2). The remaining two species (*E. griseogularis, G. melanops*) have similarly unstructured mtDNA diversity but there are no shared haplotypes among SW and SE individuals. Four SW populations and six SE populations showed signals of population expansion that reached statistical significance according to Fu's *Fs* and Ramos-Onsin and Rozas’*R*_2_ statistics (Table 3). MK tests did not reject neutrality.

**Table 1:** Polymorphism and haplotype statistics based on *ND2* sequences

Species	Sequence length	No. of individuals	No. of polymorphic sites	No. of parsimony informative sites	No. of haplotypes	Haplotype diversity HD (+SD)
*Melithreptus lunatus*	1033	30	63	55	20	0.963
						(0.945–0.981)
*Petroica boodang*	1020	38	45	32	18	0.913
						(0.887–0.939)
*Phylidonyris novaehollandiae*	1033	29	33	20	16	0.936
						(0.909–0.963)
*Malurus splendens*	987	48	38	16	22	0.75
						(0.682–0.818)
*Climacteris rufus*	1022	32	23	9	17	0.925
						(0.897–0.953)
*Gliciphila melanops*	865	27	28	16	18	0.943
						(0.911–0.975)
*Eopsaltria griseogularis*	1022	27	7	4	5	0.783
						(0.732–0.834)
*Drymodes brunneopygia*	865	14	10	3	9	0.934
						(0.889–0.979)
*Malurus pulcherrimus*	1022	10	9	1	6	0.778
						(0.641–0.915)
*Glossopsitta porphyrocephala*	1024	23	13	2	12	0.739
						(0.638–0.84)

Standard deviations (SD) are given in parentheses.

**Table 2:** Nucleotide diversity within and nucleotide divergence between SW and SE populations based on *ND2* sequences

Species	No. of individuals west	No. of individuals east	Reciprocally monophyletic?	π West (%) (SD)	π East (%) (SD)	θ-W West (SD)	θ -W East (SD)	Da (%) (SD)
*Melithreptus lunatus*	14	16	Yes	0.22	0.40	0.0027	0.0050	4.35
				(0.18–0.26)	(0.34–0.46)	(0.0014–0.0041)	(0.0029–0.0071)	(3.73–4.96)
*Petroica boodang*	10	28	Yes	0.13	0.18	0.0017	0.0038	2.85
				(0.09–0.17)	(0.16–0.21)	(0.0007–0.0027)	(0.0023–0.0053)	(2.35–3.35)
*Phylidonyris novaehollandiae*	10	19	Yes	0.27	0.33	0.0038	0.0033	1.28
				(0.21–0.34)	(0.30–0.37)	(0.0019–0.0056)	(0.0019–0.0048)	(1.04–1.53)
*Malurus splendens*	14	34	Yes	0.47	0.14	0.0052	0.0012	0.98
				(0.42–0.51)	(0.08–0.20)	(0.0039–0.0095)	(0.0033–0.0071)	(0.72–1.24)
*Climacteris rufus*	6	26	Yes	0.34	0.25	0.0039	0.0036	0.19
				(0.28–0.40)	(0.22–0.28)	(0.0017–0.0060)	(0.0021–0.0050)	(0.10–0.29)
*Gliciphila melanops*	11	16	No	0.54	0.52	0.0060	0.0085	0.08
				(0.44–0.64)	(0.45–0.59)	(0.0033–0.0087)	(0.0051–0.0119)	(0.00–0.20)
*Eopsaltria griseogularis*	12	15	No	0.05	0.0013	0.0010	0.0012	0.04
				(0.03–0.07)	(0.11–0.15)	(0.0003–0.0016)	(0.0005–0.0019)	(0.00–0.07)
*Drymodes brunneopygia*	3	11	No	0.16	0.27	0.0016	0.0032	0.03
				(0.08–0.23)	(0.24–0.31)	(0.0003–0.0029)	(0.0016–0.0048)	(0.00–0.14)
*Malurus pulcherrimus*	2	8	No	0.12	0.0021	0.0012	0.0030	0.00
				(0.06–0.18)	(0.13–0.30)	(0.0000–0.0024)	(0.0014–0.0046)	(0.00–0.12)
*Glossopsitta porphyrocephala*	9	14	No	0.23	0.06	0.0036	0.0012	0.00
				(0.19–0.28)	(0.04–0.08)	(0.0018–0.0054)	(0.0005–0.0020)	(0.00–0.05)

Nucleotide diversity is given as π (as a percentage) and Watterson's θ (θ-W). Divergence is given as net divergence, Da (as a percentage). Standard deviations (SD) are given in parentheses.

**Table 3:** Tests of population expansion

Species	Ramos and Onsin's *R*_2_ West	Ramos and Onsin's *R*_2_ East	Fu's *F* s west	Fu's *F* s east
*Melithreptus lunatus*	0.113	0.104	–1.756	–6.906
	*P* = 0.093	*P* = 0.106	*P* = 0.133	***P* = 0.002**
*Petroica boodang*	0.143	3.855	–1.547	–7.819
	*P* = 0.102	***P* = 0.001**	*P* = 0.091	***P* = 0.001**
*Phylidonyris novaehollandiae*	0.120	0.128	–3.796	–0.578
	*P* = 0.055	*P* = 0.425	***P* = 0.014**	*P* = 0.922
*Malurus splendens*	0.072	0.083	–5.995	–6.139
	***P* = 0.001**	*P* = 0.116	***P* = 0.004**	***P* = 0.015**
*Climacteris rufus*	0.099	0.093	–2.941	–4.186
	*P* = 0.0504	*P* = 0.159	*P* = 0.053	***P* = 0.025**
*Gliciphila melanops*	0.130	0.076	0.523	–4.793
	*P* = 0.177	***P* = 0.004**	*P* = 0.591	***P* = 0.019**
*Eopsaltria griseogularis*	0.144	0.158	–2.124	–0.805
	*P* = 0.147	*P* = 0.394	***P* = 0.025**	*P* = 0.222
*Drymodes brunneopygia*	0.471	0.121	NA	–2.403
	*P* = 0.310	*P* = 0.064		*P* = 0.054
*Malurus pulcherrimus*	0.500	0.173	NA	–0.965
	*P* = 1.000	*P* = 0.331		*P* = 0.216
*Glossopsitta porphyrocephala*	0.084	0.113	–5.197	–3.143
	***P* = 0.003**	***P* = 0.003**	***P* = 0.004**	***P* = 0.003**

Ramos-Onsins and Rozas's *R*_2_ and Fu's *Fs* are given with *P* values (α = 0.05) based on coalescent simulations (10,000 replicates). *P* values that reached statistical significance are in bold. NA, not applicable owing to small sample size.

### Tests for temporal congruence of divergence

**Figure 3:**
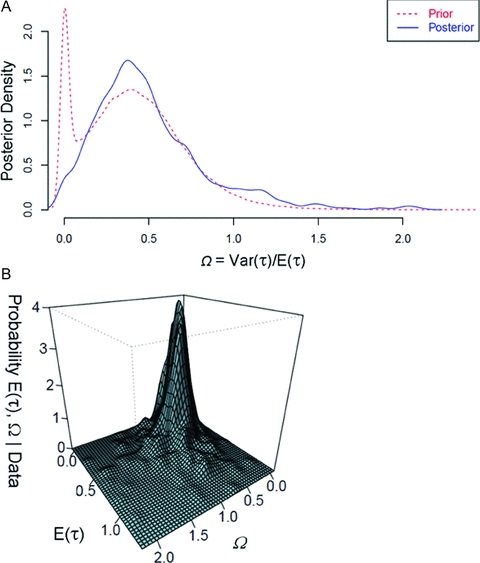
Posterior distributions of temporal concordance. (A) Prior Distribution (dashes) and posterior densities (solid line) of the number of co-divergence events, Ψ. (B) Three-dimensional diagram of the joint posterior distribution of the degree of discordance, Ω (the variance of τ, divided by the mean of τ across all population pairs), and the mean τ across all population pairs (in units of neutral mutation rate, μ) from MsBayes analyses.

Observed summary statistics from the 10 SW and SE population pairs used in MsBayes analyses are presented in Table 4. Estimates of Ω and Ψ indicated that a model of three co-divergence events was best supported by the data (Ω = 0.394; Fig. 3). The BF for the model of simultaneous divergence (Ψ = 1 vs. Ψ > 1) is 2.059, or “barely worth a mention” (Jeffreys 1961). Using threshold values of Ω = 0 versus Ω > 0.0, the BF = 0.273, indicating negative support. The BF for Ψ≤ 3 versus Ψ > 3 is 8.189, indicating a substantial level of support for no more than three co-divergence events. Notably, for Ψ≤ 5 versus Ψ > 5, the BF is 135.585, indicating a decisive level of support for no more than five divergence events. The continuous model of divergence is overwhelmingly rejected; Ψ = 10 versus Ψ < 10, BF = 3.9 × 10^−10^.

**Table 4:** Observed summary statistics from 10 co-distributed SW and SE population pairs used for acceptance/rejection in MsBayes analyses

Species	π	Net π	Watterson's θ	1/Tajima's D
*Melithreptus lunatus*	0.024787	0.041916	0.015394	0.036908
*Petroica boodang*	0.012806	0.027713	0.0105	0.032003
*Phylidonyris novaehollandiae*	0.009034	0.012496	0.008135	0.026547
*Malurus splendens*	0.007556	0.010777	0.008051	0.027008
*Climacteris rufus*	0.002571	0.002367	0.003542	0.014701
*Gliciphila melanops*	0.005678	0.000836	0.008516	0.026703
*Glossopsitta porphyrocephala*	0.001258	0.000396	0.00344	0.016245
*Eopsaltria griseogularis*	0.001143	0.000298	0.001777	0.012167
*Malurus pulcherrimus*	0.002312	0	0.00376	0.012275
*Drymodes brunneopygia*	0.002602	0	0.003686	0.014251

Fixing Ψ = 3 (the highest posterior estimate) allowed MsBayes to estimate the timing of each species assemblage divergence and to estimate the number of species that diverged at each co-divergence event. Results indicated that four SW and SE population pairs diverged more recently (mode *τ* = 8.297E-06), three diverged at mode *τ* = 0.232, and two diverged relatively early (mode *τ* = 1.145) (Table 5). The number of taxa assigned to each co-divergence event sums to nine (not 10). It is unclear whether the uncertainty lies at the boundary between the first and second co-divergence events, or between the second and third divergences. Accordingly, two of the 10 species (fifth and eighth when ranked by net π) cannot confidently be assigned to their corresponding species assemblage divergence. *Gliciphila melanops* could have diverged in either the most recent or the middle co-divergence event. *Phylidonyris novaehollandiae* could have diverged in either the deepest or the middle co-divergence event. Subsets of species were formed to account for this uncertainty in species assemblages for each co-divergence event (Table 5). Initially, *E. griseogularis, D. brunneopygia, M. pulcherrimus,* and *G. porphyrocephala* are confidently associated with the most recent co-divergence event. *Gliciphila melanops* could be associated with this event or the middle co-divergence event. Similarly, *M. splendens* and *C. rufus* are confidently assigned to the next oldest co-divergence event but *P. novaehollandiae* could be associated with this event or the oldest co-divergence event. Finally, *M. lunatus* and *P. boodang* are confidently assigned to the oldest event. We accounted for the uncertainty in the placement of *G. melanops* and *P. novaehollandiae* by analyzing all possible placements separately (see Table 5). To obtain an indication of the level of support for each possible suite of species for each co-divergence event, MsBayes analyses were repeated on all possible subsets with Ψ unconstrained to estimate Ω and Ψ (Table 5). Estimates of Ω very close to zero and *Ψ* = 1 indicate positive support, and BFs were calculated to indicate the robustness of the support (Table 5). Parameter estimates of Ω were very close to zero for all but one subset of taxa where the estimate of Ω (0.091) was a significant outlier (*P* < 0.05) (Table 5). The highest posterior probability for Ψ was equal to 1 in all but one additional subset of taxa (Table 5). This was when *M. splendens* and *C. rufus* were analyzed alone. BFs for Ψ = 1 versus Ψ >1 indicate that the assignment of species to the most recent divergence (with or without *G. melanops*), is the only co-divergence event indicating strong support for simultaneous divergence of its corresponding suites of species (BF = 27.817, 29.598, respectively). BFs for the remaining suites of species indicate support is weak. For this reason, other combinations of species were analyzed (see Flowchart, Fig. 2), for example not including both *M. splendens* and *C. rufus*. There was no increased support for these additional species combinations, possibly reflecting reduced power of analyzing only two or three species in a single species assemblage divergence.

**Table 5:** Number and timing of divergence events estimated from MsBayes analyses, assignment of species to each divergence event accounting for uncertainty, and level of support for a single divergence event for each possible suite of species

Co-divergence Event	Number of taxon pairs	Divergence Time (*τ*)		Divergence time (in years)		Possible suites of species	Ω var(*τ*)/E(*τ*)	Ψ
						
		Mode	2.5%–97.5%	Mode	2.5%–97.5%			
Tau 1	Ψ = 4	8.297E-06	0.000–0.333	10	0–396,843	*Eopsaltria griseogularis, Drymodes brunneopygia, Malurus pulcherrimus*, *Glossopsitta porphyrocephala*	0.0000	1
	1(or 5)					+*Gliciphila melanops*	0.0000	1
Tau 2	Ψ = 3	0.232	0.000–1.267	276,430	0–1,508,049	2*Malurus splendens, Climacteris rufus*	0.0130	**32**
						+*Gliciphila melanops*	**30.0910**	1
						+*Phylidonyris novaehollandiae*	0.0041	1
	1(or 4)					+*Gliciphila melanops, Phylidonyris novaehollandiae*	0.0180	1
Tau 3	Ψ = 2	1.145	0.499–2.871	1,363,215	594,382–3,417,699	*Melithreptus lunatus*, *Petroica boodang + Phylidonyris novaehollandiae*	0.0006	1
	1(or 3)						0.0020	1

1 A total of eight taxa were assigned to respective divergence events with confidence. Accounting for uncertainty, the number of taxon pairs for each divergence event is in parentheses.

2 *Malurus splendens* and *Climacteris rufus* were assigned with confidence to the second divergence event, and were analyzed alone, however the divergence event includes either *Gliciphila melanops* or *Phylidonyris novaehollandiae*, or both of these species (Psi = 3 (or 4)).

3 Ω very close to zero and Ψ = 1 indicates support for a single divergence event. A single divergence event for *Malurus splendens* and *Climacteris rufus* alone was not supported (Ψ = 2). A single divergence event for *Malurus splendens*, *Climacteris rufus* and *Gliciphila melanops* was not well supported, with Ω = 0.091 a significant outlier (*P* < 0.05).

### Estimating demographic parameters of taxon assemblage divergence

IMa analyses were performed on 10 SW and SE population pairs, divided into these same subsets of taxa. Divergence time estimates from IMa analyses are compared with divergence time estimates from MsBayes analyses and average net divergence in Table 6.

**Table 6:** Divergence time estimates from IMa analyses for all suites of species are compared with those calculated using net divergence (Da) estimates

				Divergence time (in years)		
				
Co-divergence event	Possible suites of species for species assemblage divergence analyses	Support for suite of species (msbayes analyses)	Divergence Time (*t*) IMa Analyses Peak posterior (2.5%–97.5%)	IMa Analyses Peak Posterior (2.5%–97.5%)	MsBayes Analyses Mode (2.5%–97.5%)	Calculated from average Da
Tau 1	*Eopsaltria griseogularis, Drymodes brunneopygia, Malurus pulcherrimus, Glossopsitta porphyrocephala*	Strong	0.58	56,954	10	9286
			(0.276–0.964)	(25,607–89,439)	(0–396,843)	
ψ = 4 or 5	*+ Gliciphila melanops*	Strong	0.956	94,881		15,048
			(0.284–1.844)	(26,349–171,085)		
Tau 2	^1^*Malurus splendens, Climacteris rufus*	Negative	1.164	111,119	276,430	279,048
			(0.724–4.060)	(67,172–376,685)	(0–1,508,049)	
ψ = 3 or 4	^1^(+*Gliciphila melanops* )	Negative	0.788	78,338		198,730
			(0.332–?)	(30,803–?)		
	*+Phylidonyris novaehollandiae*	Weak	2.34	228,963		389,683
			(1.604–5.748)	(148,818–533,296)		
	*+Gliciphila melanops, Phylidonyris novaehollandiae*	Weak	1.615	157,471		301,786
			(1.035–7.155)	(96,027–663,837)		
Tau 3	*Melithreptus lunatus, Petroica boodang*	Weak	9.375	869,807	1,363,215	1,713,333
			(4.625–21.625)	(429,105–2,006,355)	(594,382–3,417,699)	
ψ = 2 or 3	*+Phylidonyris novaehollandiae*	Weak	9.875	914,267		1,345,873
			(4.875–16.125)	(452,300–1,496,068)		

**Figure 4:**
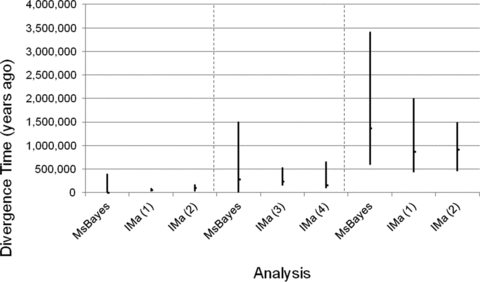
Comparison of divergence time estimates from HABC (MsBayes) and Full-Bayesian (IMa) analyses: MsBayes mode and 95 percentile and IMa peak posterior and 95% Credibility intervals for each co-divergence event. Note that Tau 2, IMa (1), and IMa (2) are not shown as they had negative support for Ψ = 1 and Ω not close to 0, respectively.

IMa peak posteriors and 95% credibility intervals were plotted alongside the MsBayes modes and 95 percentile (Fig. 4). Results were broadly consistent, but IMa results had substantially reduced credibility intervals. Peak migration rate estimates were effectively zero in all but one scenario of co-divergence. Some migration from SW to SE was inferred when *G. melanops* was assigned to the first (most recent) co-divergence event (peak posterior *m*2 = 0.825; 95% credibility interval: 0.122–3.662).

Overarching patterns of effective population sizes of ancestral, SW, and SE populations differed among the three co-divergence events. For the most recent co-divergence event, SW and SE assemblage population sizes were similar if *G. melanops* was included in the species assemblage, but if *G. melanops* was omitted the population size for the SW assemblage was very large and could not be estimated. In this instance, the model of divergence is in doubt and the corresponding divergence time should not be relied upon without the comparative context provided here. Estimates of ancestral population size were two orders of magnitude lower than current population sizes (for both SW and SE with *G. melanops* and only SE without *G. melanops*). For the middle co-divergence event, the ancestral population sizes were lower than the current population sizes, but by a more moderate scaling (2.7–5.7 times lower). Current SW and SE assemblage population sizes are similar. For the earliest co-divergence event, ancestral population size is comparable to the current population size of the SE assemblage, but the current SW population size is 1.8–4 times lower.

## Discussion

We compared demographic histories of 10 southern Australian bird species with discontinuous or patchily continuous distributions across southern Australia. Our broad aim was to explore how Plio-Pleistocene history of environmental change in southern Australia shaped evolution of the region's biota. We also wished to develop a foundation for targeting multilocus-based approaches to the same issue. Our specific aim was to use a coalescent-based HABC method, (MsBayes: Hickerson et al. 2006, 2007) to test for simultaneous divergence across one of the putative barriers in temperate southern Australia, in this case the Nullarbor Plain and its fringing semi-arid habitats (Fig. 1). Our central finding is that the model that is best supported by our data is one in which there were three separate co-divergence events in the history of the 10 SW and SE population pairs. The level of support for this model was substantial. There was only weak support for a model of simultaneous divergence and a model of continuous divergence was overwhelmingly rejected. As the distributions of our focal species are either wholly disjunct or patchily continuous across arid habitats north of the Nullarbor, we recognize the need to emphasize the distinction between co-divergence events and which barriers to gene flow are associated with them. The three temporal co-divergences may have been associated not just with the Nullarbor Plain but also with barriers other than the Nullarbor Plain and its fringing semi-arid habitats (e.g., Eyrean Barrier; see Introduction). Issues arising from the simple conclusion are now addressed.

Regarding the assignment of species to particular co-divergence events, it is of interest to note that the two assigned to the earliest divergence event (*M. lunatus*, *P. boodang*) are restricted to more mesic habitats (predominantly wetter open forest and woodland formations). These are the habitats that would have been isolated longest across southern Australia (Byrne 2008; Byrne et al. 2011). Indeed, our results are consistent with and offer some independent affirmation of Toon et al.'s (2010) conclusion that the eastern and western subspecies of *M. lunatus* that were recognized when this work began actually are two non-sister taxa, *M. lunatus* in the SE and *M. chloropsis* in the SW. *Phylidonyris novaehollandiae* may have diverged early with *P. boodang* and *Melithreptus* species, or in the middle divergence event. This species certainly inhabits open forest and woodland, but is also common in drier heaths. The remaining seven species are more tolerant of drier, semi-arid habitats.

Broad consistency of divergence time estimates derived from MsBayes and IMa is promising. This is all the more so because MsBayes is specifically designed for testing simultaneous divergence of multiple taxa across a common barrier, whereas IMa is designed for multilocus inference within a single population split. To our knowledge, this is the first time IMa has been applied to an assemblage of species. What made it possible was that MsBayes can first identify the different temporal co-divergence events prior to performing IMa on appropriate species assemblages. Assuming adequate performance of the inheritance scalar in accounting for different effective population sizes between SW and SE population pairs, inference from IMa analyses resulted in reduced credibility intervals around divergence time estimates for each co-divergence event. As noted earlier, this approach assumes that effective population sizes of the ancestral and the two divergent daughter populations vary consistently with respect to each other across all species. The extent to which this assumption will affect inference from IMa analyses is yet to be tested. Caution is especially needed when drawing conclusions on effective population sizes. In MsBayes analyses, these parameters are intentionally decoupled across taxa for this purpose. So the broad congruence of the divergence time parameter between MsBayes and IMa provides a level of confidence that this assumption is not greatly affecting the model of divergence of the species assemblage in the IMa analyses in this case presented here.

### Converting divergence time estimates to a demographic scale

Converting divergence time to parameters on a demographic scale warrants some comment. The issue is contentious given uncertain estimations of mutation rates (Ho 2007). In addition, applying a mutation rate generated from a phylogeny estimated using a GTR +Γ nucleotide substitution model to a population divergence model using a HKY nucleotide substitution model (as in IMa) is likely to underestimate divergence time (Arbogast et al. 2002). Given these limitations, the divergence time estimates have been converted to a demographic scale to make divergence time parameters broadly comparable across analyses and to provide only a very coarse timescale for these species assemblage divergences.

According to the IMa analysis, which requires no assumption of generation time, the deepest divergence of two or three mesic SW and SE population pairs is from the middle to the early Pleistocene (95% CI: 429,105–2,006,355 years). Consistent with the potential for the HKY nucleotide substitution model to underestimate divergence time, especially in older divergences, this estimate is on the lower limit of Toon et al.'s (2010) divergence time for SW *M. chloropsis* and SE *M. lunatus* (2–4.6 Mya) from relaxed molecular-clock dating. This divergence may be consistent with the progression of aridification following the step at 1.4–1.5 Mya identified by McLaren and Wallace (2010). In contrast, the most recent divergence of semi-arid SW and SE population pairs (95% CI: 25,607–171,085) is bound at the upper limit at the mid-Pleistocene, and includes the Last Glacial Maximum. Pertinent to this time period is that plant species with more palatable leaves and fleshy fruits were present in the region of the Nullarbor between 180,000 and 400,000 years but are now restricted to remnant stands on the Nullarbor Plain's fringes (Prideaux et al. 2007). The limit for the most recent divergence is consistent with the floristic transition to present-day's dominant treeless chenopod shrubland habitats. Similarly, Hocknull et al. (2007) noted extinction of mesic fauna in central eastern Australia within this timeframe.

Interspecific divergence times of 23 sister species pairs of plants endemic to either side of the Nullarbor Plain clustering around 13–14 Mya (Crisp and Cook 2007) are relatively ancient compared to the three divergence times identified here in an assemblage of birds. Mid-Miocene aridification and/or elevation of the Nullarbor Plain as a vicariant barrier were inferred as the driving agents of the more ancient divergences. In Crisp and Cook's (2007) study, three younger divergences of 2–4 Mya are consistent with the deepest of the three divergence events in our study. This cluster of divergences was thought to be consistent with severe drying around this time (Fujioka et al. 2005).

Synthesizing observations of population size from IMa results, we suggest they are consistent with species being most dramatically affected prior to (or around the time of) the most recent co-divergence event (bound at the upper limit at the mid-Pleistocene, and including the Last Glacial Maximum). This is consistent with the floristic transition to the present-day's dominant treeless chenopod shrubland habitats (discussed above). Perhaps not unexpectedly, the methodology used here supports this simple conclusion but in so doing suggests the utility of the method more generally.

Despite the potential for gene flow in times of lower sea level during glacial maxima across land now submerged under the present day Great Australian Bight (Haq et al. 1987; Macphail et al. 1994; Li et al. 2004), there was generally no evidence for migration. Migration was inferred only for the most recent co-divergence event and indeed only when *G. melanops* was included. In this case, there was postdivergence gene flow from populations of the SW taxon assemblage to populations of the SE taxon assemblage.

## Conclusions

Our central finding is that 10 bird species with discontinuous or patchily continuous distributions across southern Australia were differentially affected by three Plio-Pleistocene divergence events. The youngest two of the three co-divergence events are clearly within the Pleistocene, but the oldest potentially reaches back to the Pliocene according to the 95 percentile of MsBayes results. It may be argued that we have not tested for the specific locations of these co-divergence events but the Nullarbor Plain and its fringing habitats are plausibly invoked as a barrier wholly or in part for each species assemblage divergence. The Eyrean Barrier to the east may also have contributed, especially to the earliest co-divergence event. This simple conclusion provides the basis for future studies on the biogeographic influence of Plio-Pleistocene environmental change in southern Australia, as well as for future multilocus-based approaches to the same issue. Our study highlights the synergistic benefits of jointly applying tests for simultaneous divergence with coalescent-based analyses of divergence. HABC methods (MsBayes) were first used to identify species assemblages shown to have simultaneously diverged, and then we applied a full Bayesian coalescent-based model of divergence (using IMa) in order to estimate parameters associated with co-divergence of this species assemblage. Divergence time estimates from MsBayes were broadly consistent with those from IMa applied to the divergence of species assemblages. Application of IMa in this context also enables exploration of migration and effective population sizes among co-diverging species assemblages. The joint application we have suggested of these methods utilizes the power of species assemblages to improve the insight that can be gained from single-locus data.

## Supporting Information

**Table S1.** Two hundred forty-six *ND2* sequences, collected from the 10 species, submitted to GenBank for individual level identification.
